# Microstructure and Optical Properties of E-Beam Evaporated Zinc Oxide Films—Effects of Decomposition and Surface Desorption

**DOI:** 10.3390/ma13163510

**Published:** 2020-08-09

**Authors:** Lukasz Skowronski, Arkadiusz Ciesielski, Aleksandra Olszewska, Robert Szczesny, Mieczyslaw Naparty, Marek Trzcinski, Antoni Bukaluk

**Affiliations:** 1 Institute of Mathematics and Physics, UTP University of Science and Technology, Kaliskiego 7, 85-796 Bydgoszcz, Poland; aleksandra.lewandowska@utp.edu.pl (A.O.); mieczyslaw.naparty@utp.edu.pl (M.N.); marek.trzcinski@utp.edu.pl (M.T.); antoni.bukaluk@utp.edu.pl (A.B.); 2 Faculty of Physics, University of Warsaw, Pasteura 5, 02-093 Warsaw, Poland; Arkadiusz.Ciesielski@fuw.edu.pl; 3 Faculty of Chemistry, Nicolaus Copernicus University in Torun, Gagarina 7, 87-100 Torun, Poland; robert.szczesny@umk.pl

**Keywords:** ZnO, decomposition, segregation, annealing, optical constants, microstructure, e-beam

## Abstract

Zinc oxide films have been fabricated by the electron beam physical vapour deposition (PVD) technique. The effect of substrate temperature during fabrication and annealing temperature (carried out in ultra high vacuum conditions) has been investigated by means of atomic force microscopy, scanning electron microscopy, powder X-ray diffraction, X-ray photoelectron spectroscopy and spectroscopic ellipsometry. It was found that the layer deposited at room temperature is composed of Zn and ZnO crystallites with a number of orientations, whereas those grown at 100 and 200 ${}^{\circ }$C consist of ZnO grains and exhibit privileged growth direction. Presented results clearly show the influence of ZnO decomposition and segregation of Zn atoms during evaporation and post-deposition annealing on microstructure and optical properties of zinc oxide films.

## 1. Introduction

Zinc oxide exhibits high thermal and chemical stability and has been studied for decades [1,2]. ZnO is a semiconductor with a direct band-gap of $E_{g}∼$ 3.3 eV [3,4] similar to the value reported to gallium nitride ($E_{g}∼$ 3.4 eV) [5], however the binding energy of zinc oxide exciton (60 meV) [4] is 2.4 times larger than that of GaN (25 meV) [5]. These features indicate that ZnO is a promising material for short-wavelength optoelectronic devices such as light-emitting and laser diodes [6]. Due to good optical transmittance in the visible spectral range zinc oxide is used as a transparent electrode in organic and hybrid solar cells [7,8]. Moreover, ZnO is used in thin-film transistors [9] and gas sensors [10]. Zinc oxide is a very good candidate for space applications due to its stability to high energy radiation [1]. On the other hand, ZnO can be easily etched in acids and alkali [11]. This feature is commonly used to produce ZnO in form of nanosheeds, nanoshells, multipods, nanorods or nanowires typicaly implemented as periodic structures using a number of methods [2,12].

The structural and optical properties of ZnO films strongly depend on the fabrication conditions that affect the optimal performance of the device. It has been shown that optical constants (complex refractive index or complex dielectric function) of zinc oxide demonstrate significant film-thickness relationship, especially for thicknesses below 20 nm [3,13,14,15]. Moreover, Pal et al. [3] revealed the influence of the substrate on the optical properties of ZnO films. Therefore, systematic study of the properties of zinc oxide layers fabricated under various growing conditions is desirable. This analysis was performed for ZnO produced by different techniques such as atomic layer deposition [3,4,9], electron beam evaporation [16,17,18,19], magnetron sputtering [10,19,20], chemical vapour deposition [6], spin coating [21,22] as well as molecular beam epitaxy [23]. In addition, the existence of oxygen or zinc vacancies and their concentration in ZnO, significantly affects luminescence and photocatalytic properties [22], depending on the method of production.

Electron beam physical vapour deposition (e-beam PVD) technique is commonly used to produce both metallic [24,25,26] and oxide coatings [27] including zinc oxide films [16,19]. The influence of microstructure on the optical response of zinc oxide films fabricated under various conditions, such as the substrate temperature (and/or annealing after deposition) as well as deposition rate, has been investigated in recent years [16,18,19]. It has been reported that increasing the substrate temperature and/or annealing of ZnO after deposition significantly improves their stoichiometry, resistivity, free carrier mobility and optical transparency [16,18]. The post-evaporation annealing process in oxygen atmosphere also serves to ensure that there is no deficiency of oxygen atoms in the deposited films [19], due to decomposition and release oxygen into the vacuum [28,29,30,31]. This process was also observed for ZnO films [19,20].

In this paper we show the influence of substrate temperature during deposition by means of the e-beam technique and annealing in ultra high vacuum (UHV) conditions on the microstructure and optical properties of zinc oxide films. The above relationship was studied based on the results of atomic force microscopy (AFM), scanning electron microscopy (SEM), X-ray diffraction (XRD), X-ray photoelectron spectroscopy (XPS) and spectroscopic ellipsometry (SE) measurements. The above-mentioned methods were used to explain the influence of ZnO decomposition and segregation of Zn atoms on the optical response of the deposited films.

## 2. Materials and Methods

Fabrication of the samples has been conducted using the PVD75 e-beam evaporation system from Lesker (St. Leonards-on-Sea, UK). ZnO films have been deposited from 4 N ZnO pieces in graphite crucible onto polished Si substrates (100). Silicon is widely used in fabrication of a variety of optical devices (e.g., [32,33]). Substrates were located 40 cm away from the crucible. Three sets of samples have been fabricated—with the substrate at room temperature (RT), at 100 and 200 ${}^{\circ }$C. The base pressure was $2\times 10^{-5}$ Torr, however, due to the decomposition of ZnO, during the deposition process the pressure approached $10^{-4}$ Torr. The deposition rate and total film thickness were monitored by two quartz weights inside the deposition chamber. The nominal deposition rate was 0.3 Å/s, however, due to the decomposition of ZnO, it is difficult to estimate the true value (although it is expected to be within 30% of the nominal value). After the deposition, the samples were annealed for two hours at 300, 500 and 800 ${}^{\circ }$C. The annealing was performed in a UHV preparation chamber (base pressure $\leq 5\times 10^{-10}$ mbar). The list of prepared samples is summarized in Table 1.

After cooling to room temperature, the samples were transferred without venting to the analysis chamber (base pressure $\leq 2\times 10^{-10}$ mbar) for XPS measurements. The incident radiation was produced by Al K${}_{\alpha }$ source (1486.6 eV) at 55 degrees with respect to the normal of the sample. The energy of photoelectrons was analyzed by the VG-Scienta R3000 (Uppsala, Sweden) spectrometer (the energy step was set at ΔE = 0.1 eV. For quantitative analysis, the experimental data was fitted to Gauss–Lorentz shapes by using CasaXPS software (version 2.3.16, Casa Software Ltd., Teignmouth, UK).

The surface topography of the films was examined by means of the AFM Innova device from Bruker (Billerica, MA, USA) equipped with the standard Si tips for a tapping mode. Roughness parameters $R_{a}$ (the arithmetical mean deviation):

(1) $$R_{a}=\frac{1}{N}\sum_{j=1}^{N}\left|Z_{j}\right|$$

and $R_{q}$ (the root mean squared roughness):

(2) $$R_{q}=\sqrt{\frac{1}{N}\sum_{j=1}^{N}Z_{j}^{2}}$$

where $Z_{j}$ and *N* are the current surface height value and the number of points measured, respectively. They were calculated based on the images, recorded for an area 2 μm × 2 μm, using the NanoScope Analysis software (version 1.40). Scanning electron microscopy (SEM) investigations were performed with a Quanta 3D FEG (FEI, Hillsboro, OR, USA) (EHT = 30 kV) device.

The Phillips X’Pert (Malvern Panalytical Ltd., Malvern, UK) device with Cu K${}_{\alpha }$ radiation (λ = 1.5418 Å) and X’Celerator Scientific (Malvern Panalytical Ltd., Malvern, UK) detector was used to record the XRD patters of the investigated samples. These measurements were performed in the range from 2θ = 20 ${}^{\circ }$ to 60 ${}^{\circ }$.

The thickness and optical constants of the prepared thin films were investigated by means of the V-VASE device from J.A. Woollam Co., Inc., Lincoln, NE, USA. The ellipsometric azimuths Ψ and Δ were measured for three angles of incidence (65${}^{\circ }$, 70${}^{\circ }$ and 75${}^{\circ }$) in the NIR-vis-UV spectral range (193–2000 nm; 0.6–6.5 eV). The analysis of ellipsometric data was performed using the WVASE32 software (J.A. Woollam Co., Inc.).

## 3. Results

**Figure 1:**
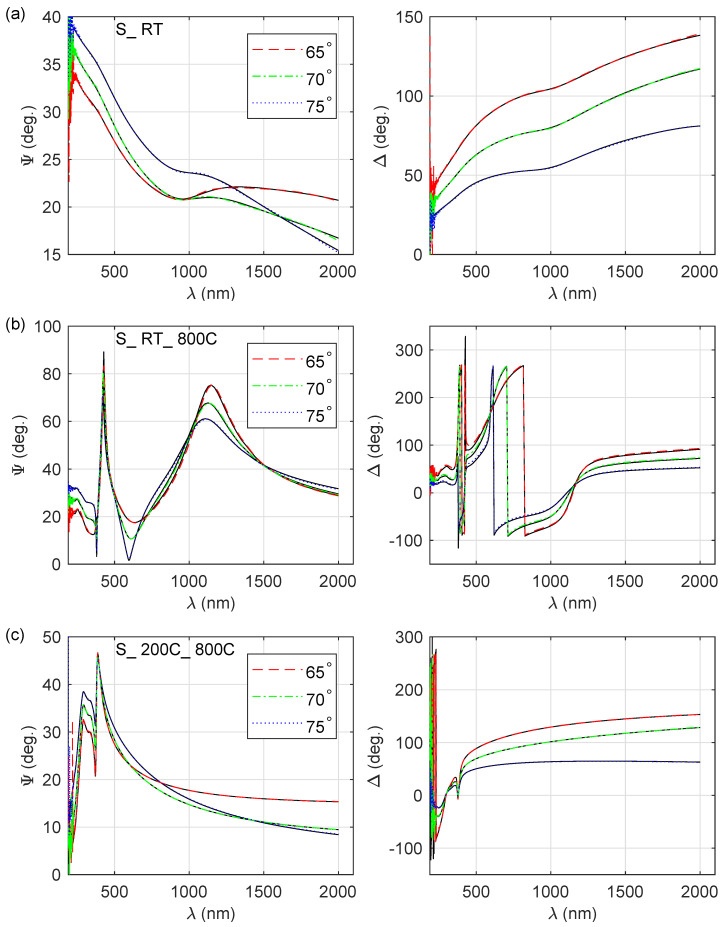
Experimental and calculated ellipsometric azimuths Ψ and Δ for (**a**) S_ RT ($\chi^{2}$ = 2.233), (**b**) S_ RT_ 800 ($\chi^{2}$ = 2.242) and (**c**) S_ 200C_ 800C ($\chi^{2}$ = 2.57) samples.

Spectroscopic ellipsometry (SE) was used to obtain the thickness of the zinc oxide film as well as the optical constants of the deposited coatings. The measured Ψ and Δ ellipsometric azimuths for S_ RT, S_ RT_ 800C and S_ 200C_ 800C are presented in Figure 1. The Ψ and Δ curves vary significantly for films fabricated in different conditions. Spectra shown in Figure 1a exhibit metallic-like behaviour, while data presented in Figure 1b,c are typical for a semiconducting material. Oscillations in the ellipsometric Ψ and Δ angles for wavelengths longer than about 390 nm for the S_ RT_ 800C sample (Figure 1b) indicate that the thickness of the layer is significantly greater compared to the thickness of S_ 200C_ 800C film. The thickness of the layer and its optical constants were determined by means of the five-medium optical model of a sample (from bottom to top: substrate-Si∖native SiO${}_{2}$∖ZnO film∖rough layer∖ambient). The complex refractive index of the ZnO film ($\tilde{n}=n+ik$, where *n* and *k* are the real part of $\tilde{n}$ and the extinction coefficient, respectively) was parametrised using a model containing the following components/oscillators:

- a Sellemeier-type dispersion relation (a Pole oscillator; $\varepsilon_{P}$),
- a Drude term (${\tilde{\varepsilon }}_{D}$; for the S_RT and S_ RT_ 300 samples) or a Tauc–Lorentz oscillator (${\tilde{\varepsilon }}_{T_{L}}$; for the other specimens) and
- a sum of Gaussian oscillators (${\tilde{\varepsilon }}_{G}$).

The selection of these oscillators was performed based on the shape of the measured ellipsometric azimuths (see Figure 1). The complex refractive index of the deposited film can be written in the following form:

(3) $${\tilde{n}}^{2}=\varepsilon_{\infty }+\varepsilon_{P}\left(A_{0},E_{0}\right)+{\tilde{\varepsilon }}_{T_{L}}\left(A_{T-L},E_{g},E_{n},Br_{T_{L}}\right)+\sum_{j}{\tilde{\varepsilon }}_{G}\left(A_{j},E_{j},Br_{j}\right)$$

or

(4) $${\tilde{n}}^{2}=\varepsilon_{\infty }+\varepsilon_{P}\left(A_{0},E_{0}\right)+{\tilde{\varepsilon }}_{D}\left(\hbar \omega_{P},\hbar \Gamma \right)+\sum_{j}{\tilde{\varepsilon }}_{G}\left(A_{j},E_{j},Br_{j}\right).$$

In Equations (3) and (4), $\varepsilon_{\infty }$ is the high-frequency dielectric constant, $E_{0}$ and $A_{0}$ are the pole oscillator position and magnitude, respectively, while $\hbar \omega_{P}$ is the unscreened plasma energy and ℏΓ is the free-carrier dumping. The quantities *A*, *E* and Br (with adequate subscripts) represent amplitude, energy and broadening of the line shape, respectively, while $E_{g}$ is the band gap energy. Mathematical formulas for the particular line shapes can be found elsewhere [34,35]. The form of $\tilde{n}$ (Equation (3) or Equation (4)) used to determine optical constants of the produced films are summarized in Table 2.

The complex refractive index of Si and SiO${}_{2}$ were employed from the database of optical constants [35]. The thickness of native oxide was set to be 2.3 nm and was established in the separate experiment [36]. The imperfect surface of the deposited film (the rough layer) was described based on the Bruggeman Effective Medium Approximation (EMA) model [34,35]. In this approach, the optical constants of the rough film are represented as a combination of optical properties of ZnO film and ambient (the assumed volume fraction of each medium was set to be 1/2). The model quantities were minimized to reduce the standard mean squared error ($\chi^{2}$) [35]:

(5) $$\chi^{2}=\frac{1}{N-P}\sum_{j}\left(\frac{{\left(\Psi_{j}^{mod}-\Psi_{j}^{exp}\right)}^{2}}{\sigma_{\Psi \;j}^{2}}+\frac{{\left(\Delta_{j}^{mod}-\Delta_{j}^{exp}\right)}^{2}}{\sigma_{\Delta \;j}^{2}}\right).$$

In Equation (5) *N* and *P* are the total number of data points and the number of fitted model parameters respectively. The $\Psi_{j}$ and $\Delta_{j}$ represent measured (with the superscript ‘exp’) or calculated (with the superscript ‘mod’) ellipsometric azimuths. Quantities $\sigma_{\Psi \;j}$ and $\sigma_{\Delta \;j}$ are standard deviations for measured Ψ and Δ angles. Example of the fits are presented in Figure 1. It should be noted that the reduced mean squared error ($\chi^{2}$) was below 2.57 for all the samples produced, which means that the calculated Ψ and Δ values are in a very good agreement with the experimental data.

**Figure 2:**
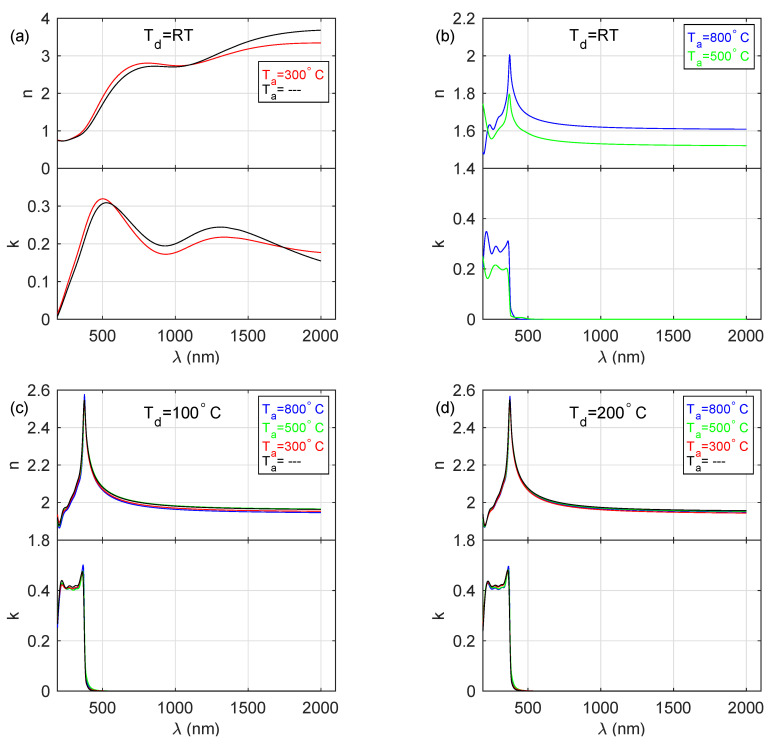
The index of refraction (*n*) and the extinction coefficient (*k*) for the layers deposited at (**a**) and (**b**) room temperature, (**c**) 100 ${}^{\circ }$C and (**d**) 200 ${}^{\circ }$C and annealed at 300, 500 and 800 ${}^{\circ }$C.

The thicknesses of the prepared films ($d_{ZnO}$) are in the range from about 37 to 325 nm, while thicknesses of the rough layer ($d_{r}$) were found to be 5–41 nm (see Table 2), whereas for the samples deposited at room temperature these values are one order of magnitude higher, than those obtained for the specimens produced at higher temperatures. The real part (*n*) of the complex refractive index ($\tilde{n}$) and the extinction coefficient (*k*) determined by the spectroscopic ellipsometry method are presented in Figure 2. The spectra shown in Figure 2a (for S_ RT and S_ RT_ 300C samples) exhibit metallic-like behaviour (noticeable extinction coefficient in the UV-vis-IR), however without the strong Drude contribution characteristic for conductors. Plasma energy ($\hbar \omega_{P}$), found during the analysis of the SE data, established to be 1.7 and 1.8 eV for S_ RT_ 300C and S_ RT samples, respectively, is much lower than that reported for pure zinc (10 eV) [37]. The *n* and *k* spectra presented in Figure 2b–d are typical for semiconducting materials, i.e., a normal dispersion for longer wavelengths and interband transitions in the high-energy region.

The band-gap energy ($E_{g}$) for the produced ZnO films was determined during the parametrisation of their optical constants (a Tauc–Lorentz oscillator; see Equation (3)) and are summarized in Table 3. Values of $E_{g}$ were established to be 3.2 eV for the layers deposited at 100 and 200 ${}^{\circ }$C as well as at RT (however only for films annealed at 500 and 800 ${}^{\circ }$C) and are in a perfect agreement with published data [3,4,13,15,38]. For metallic-like samples (S_RT and S_RT_300C) the band-gap energy could not be determined.

**Figure 3:**
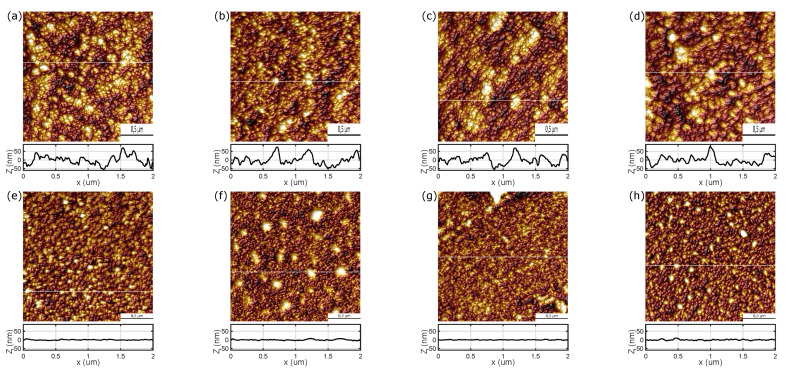

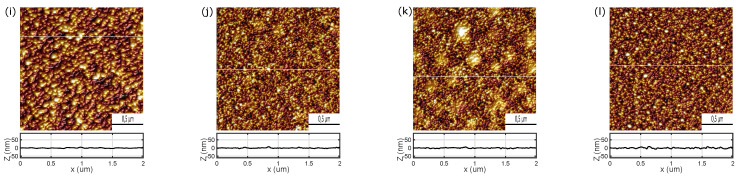
AFM images of ZnO layers deposited at RT: (**a**) not annealed and annealed at (**b**) 300 ${}^{\circ }$C, (**c**) 500 ${}^{\circ }$C and (**d**) 800 ${}^{\circ }$C and deposited at 100 ${}^{\circ }$C: (**e**) not annealed and annealed at (**f**) 300 ${}^{\circ }$C, (**g**) 500 ${}^{\circ }$C and (**h**) 800 ${}^{\circ }$C as well as deposited at 200 ${}^{\circ }$C: (**i**) not annealed and annealed at (**j**) 300 ${}^{\circ }$C, (**k**) 500 ${}^{\circ }$C and (**l**) 800 ${}^{\circ }$C.

**Figure A1:**
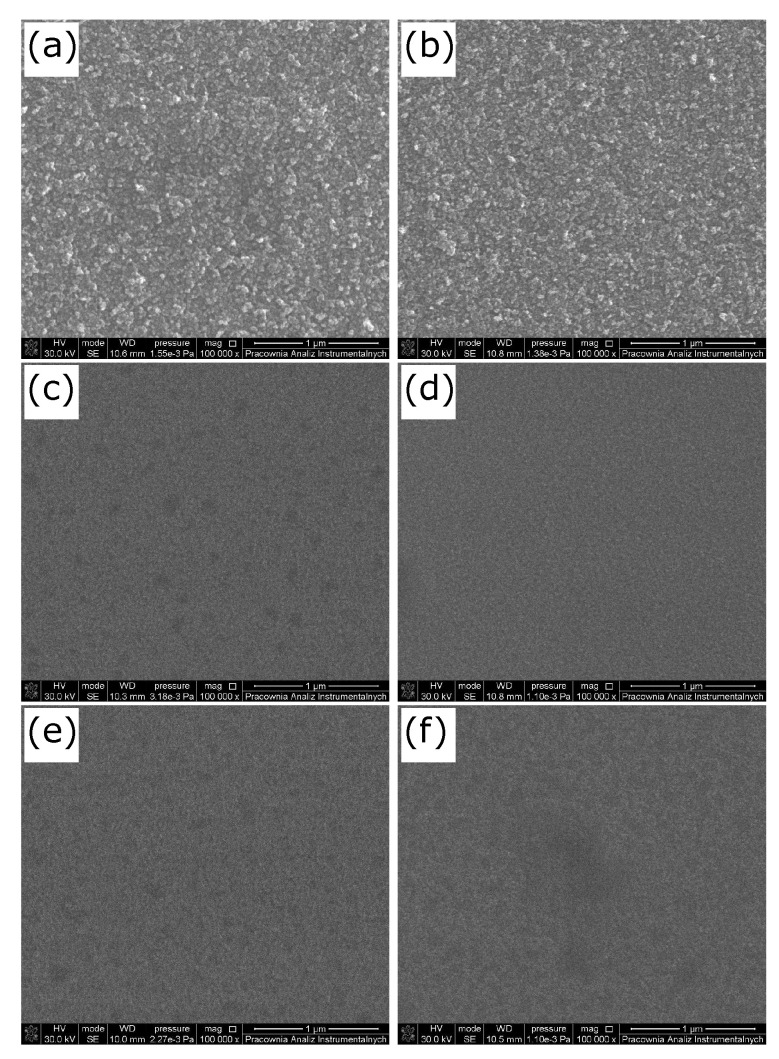
Scanning electron microscopy (SEM) images of ZnO layers deposited at RT: (**a**) not annealed and (**b**) annealed at 500 ${}^{\circ }$C, deposited at 100 ${}^{\circ }$C: (**c**) not annealed and (**d**) annealed at 500 ${}^{\circ }$C as well as deposited at 200 ${}^{\circ }$C: (**e**) not annealed and (**f**) annealed at 500 ${}^{\circ }$C.

AFM images of the ZnO films deposited at RT, at 100 ${}^{\circ }$C and at 200 ${}^{\circ }$C (both non-annealed and annealed after deposition) are presented in Figure 3. Surfaces of all samples exhibit the nanogranular structure with the lateral size of grains smaller than 100 nm (for specimens produced at RT; Figure 3a) or 50 nm (for the other surfaces; Figure 3b,c). It should be taken into account that the samples deposited at RT exhibit significantly expanded surface compared to the surface of the other ZnO layers (see profiles in Figure 3). The surface morphology of the deposited films has been confirmed in the SEM measurements (see Figure A1 in Appendix A). For ZnO films deposited at RT the average roughness ($R_{a}$) is about 20 nm (see Table 2) and the maximum roughness ($R_{max}$) 150–160 nm, whereas for the zinc oxide layers grown at higher temperatures (100 and 200 ${}^{\circ }$C) these values are significantly lower ($R_{a}$ = 1.5–2.9 nm; $R_{max}$ = 11–32 nm).

**Figure 4:**
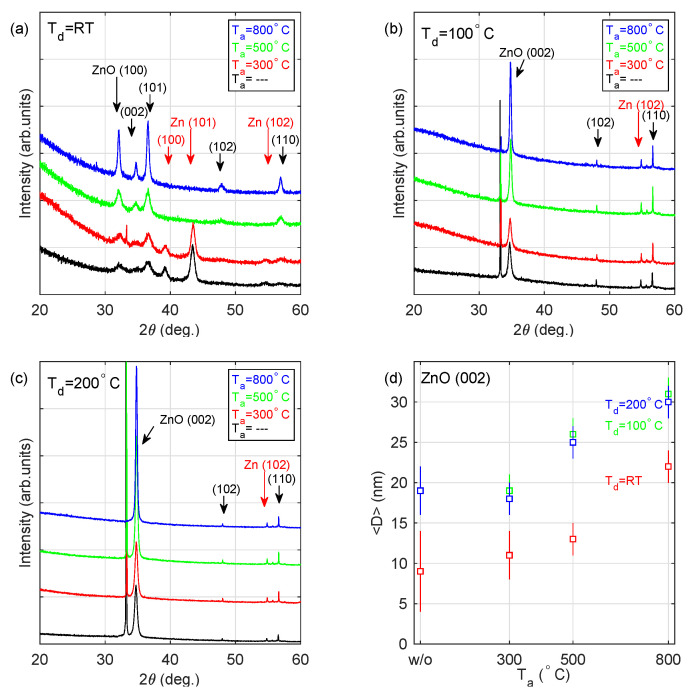
XRD patterns for the samples deposited at (**a**) room temperature, (**b**) 100 ${}^{\circ }$C and (**c**) 200 ${}^{\circ }$C and annealed at 300, 500 and 800 ${}^{\circ }$C. (**d**) The average size (<D>) of the ZnO(002) crystallite. Used ICDD cards: Zn-00-001-1238 and ZnO-00-001-1136.

The XRD patterns recorded for the deposited coatings are presented in Figure 4. Diffraction peaks related to ZnO are observed at 2θ≈32${}^{\circ }$ (100), 34${}^{\circ }$ (002), 36${}^{\circ }$ (101), 48${}^{\circ }$ (102) and 57${}^{\circ }$ (110), while the signal recorded at 2θ≈39${}^{\circ }$ (100), 43${}^{\circ }$ (101) and 55${}^{\circ }$ (102) can be referred to metallic zinc. The strong and narrow peak at 2θ≈33${}^{\circ }$ can be assigned to the substrate-Si (200). The average crystallite size (<D>) determined for the identified Zn and ZnO phases, summarized in Table 4, has been estimated based on the Scherrer formula:

(6) $$<D>=\frac{0.94\lambda }{\beta \cos\left(2\theta \right)}$$

where λ is the X-ray wavelength and β is the full-width at half-maximum (FWHM) of the Bragg diffraction peak at angle 2θ.

The layer deposited at RT (a sample S_ RT; see Figure 4a) consists of both ZnO and Zn crystallites, which are relatively small (<D> = 6–13 nm) and show a number of orientations. Annealing of the deposited film at 300 ${}^{\circ }$C (a sample S_ RT_ 300C) does not change the phase composition of the coating. The layers annealed at higher temperatures ($T_{a}\geq$ 500 ${}^{\circ }$C; samples S_ RT_ 500C and S_ RT_ 800C) contain only ZnO grains. The most prominent XRD peaks (2θ = 30${}^{\circ }$–40${}^{\circ }$) for these two samples are narrower than that recorded for S_ RT and S_ RT_ 300C—the average crystallite size is in the range 11–15 nm and 12–25 nm for S_ RT_ 500C and S_ RT_ 800C films, respectively. It should be noted that no growth direction is privileged. Completely different XRD patterns have been registered for samples deposited at 100 and 200 ${}^{\circ }$C (see Figure 4b,c). Those films are composed of only ZnO (200) crystallites (the other XRD peaks show negligible intensity). The registered (200) direction of growth (as provileged orientation) was observed earlier for ZnO layers produced using various methods [14,17,19]. The average crystallite size increases with the increase in the annealing temperature from about 19 to about 30 nm (see Figure 4d).

**Figure 5:**
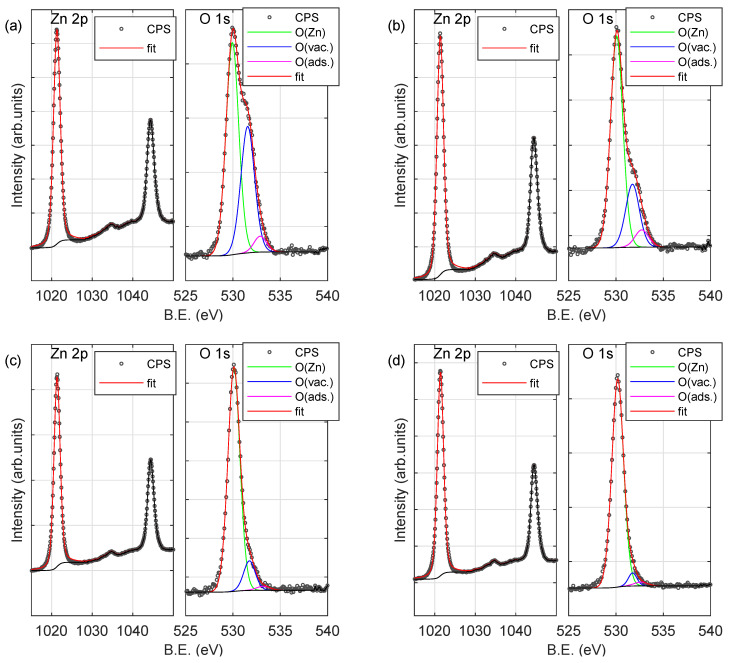
X-ray photoelectron spectroscopy (XPS) spectra (Zn 2p and O 1s peaks) of the sample deposited at room temperature (RT): (**a**) non-annealed and annealed at (**b**) 300 ${}^{\circ }$C, (**c**) 500 ${}^{\circ }$C and (**d**) 800 ${}^{\circ }$C.

To investigate the chemical state of the surface of the produced samples the Zn 2p, O 1s and C 1s peaks have been recorded. Figure 5 shows Zn 2p and O 1s XPS signals for samples deposited at room temperature (both non-annealed and annealed at 300, 500 and 800 ${}^{\circ }$C). The spectrum of zinc exhibits a characteristic doublet at 1022 eV (2p 3/2) and 1045 eV (2p 1/2) [14,20,39]. The O 1s signal consists of three components. The most prominent (with maximum at 530 eV; O(Zn)) can be assigned to oxygen atoms in the ZnO compound [14,20,21,40,41]. The second one (at 531 eV; O(vac.)) is associated with the O${}^{2-}$ in the oxygen-deficient region (vacancies) within the matrix of zinc oxide [42,43]. The last one (at 532 eV; O(ads.))—is connected to the adsorbed oxygen at the surface of the specimen [41]. It should be noted that the intensity (and thus concentration) of the second and the third components of the O 1s peak significantly decreases with the increase of the annealing temperature. Concentrations of particular elements are summarized in Table 5. The concentration of Zn (C${}_{Zn}$) and O bounded with Zn (C${}_{O(Zn)}$) atoms increases with the increase in the annealing temperature (for all series of samples). The opposite can be observed for the adsorbed oxygen (C${}_{O(ads.)}$) and oxygen ions (O${}^{2-}$) in the oxygen-deficient region as well as carbon (related to both sp${}^{3}$ hybridization and adsorbed at the surface as CO${}_{2}$) [39]. Despite the fact that the concentration of carbon (C${}_{C(tot.)}$) for the non-annealed sample is 12–15% (see Table 5), it decreases rapidly with increasing annealing temperature and is about 1% for samples annealed at 800 ${}^{\circ }$C.

Deconvolution of the O 1s signal allowed us to determine the ratio of Zn and O atoms at the surface of the prepared films taking into account total concentration of oxygen (C${}_{O(tot.)}$) or the part related only to O (C${}_{O(Zn)}$) atoms bounded with Zn atoms. These estimated ratios are presented in Table 6. The ratio between zinc and oxygen atoms (total) for the non-annealed samples (S_ RT, S_ 100C and S_ 200C) is about 1 and increases to about 1.6 with the increase of the annealing temperature. Significantly higher values have been determined taking into account only the part of O 1s peak related to oxygen atoms bounded with zinc atoms. These values are in the range from about 1.5 to 1.9 (see Table 6).

## 4. Discussion

Both AFM (see Figure 3 and Table 2) and XRD (see Figure 4 and Table 4) results show that the grain size perpendicular to the surface increases with annealing temperature as the grains coalesce, regardless of the sample deposition temperature. The trend is similar for the lateral grain size, however in the case of ZnO deposited at RT the lateral grain size is already so large that no annealing-induced coalescence is expected.

Further results presented in the previous section unambiguously show that the main changes during annealing were observed for the samples deposited at room temperature. The changes were much less prominent for samples deposited at elevated temperatures. Therefore in this section, we focus our attention on the explanation of this phenomenon and on the effect of annealing on microstructure and optical properties of the fabricated films.

First, it was noticed that the thicknesses of ZnO films deposited onto substrates at elevated temperatures during the deposition process (100 and 200 ${}^{\circ }$C) as determined form ellipsometric measurements are significantly lower (by an order of magnitude) than in the case of films deposited onto substrates at room temperature (see Table 2). Second, as described in the Results section, the permittivity of these films exhibits a more oxide-like behaviour (Figure 2b–d), whereas films deposited onto substrates at room temperature have more metal-like characteristics (Figure 2a). This might be a consequence of two phenomena—ZnO decomposition during the deposition process (which will result in an excess of Zn atoms in the deposited film) as well as subsequent excess Zn atom surface segregation as desorption in elevated temperatures, which would improve stoichiometry. The details are described below.

During the e-beam evaporation process, many oxide materials are known to decompose [28,29,30,31] releasing oxygen into the vacuum. The decomposition of ZnO has also been observed [19,20]. Thus, to ensure there is no deficiency of oxygen atoms in the evaporated film, it has been proposed to perform the deposition process in an oxygen atmosphere [19]. In our case, the ZnO films have been deposited in a high vacuum. This approach results in the evaporated films containing an excess of Zn atoms with respect to typical ZnO stoichiometry. Since samples fabricated using e-beam technology are typically polycrystalline, the excess Zn atoms are expected to segregate to the ZnO grain boundaries as well as to the film’s free surface, as this process would result in the release of the elastic strain energy accumulated in the non-stoichiometric ZnO crystallites [44,45]. This is due to the fact that within the crystallites there is a limited number of lattice vacancies which enable the excess Zn atoms to occupy substitutional lattice sites. Most of the excess Zn atoms are therefore expected to occupy the interstitial lattice sites. This would induce much higher local distortions (strain) to the crystal lattice. Because of numerous lattice vacancies being present on the free surface and at the grain boundaries, there are more substitutional lattice sites in those regions for the excess Zn atoms to occupy. Moreover, the excess Zn atoms present in the interstitial sites on the surface and at the grain boundaries induce strain in a lower volume of the crystal lattice than the excess Zn atoms present in the interstitial lattice sites inside the ZnO grains [44,45]. Although elastic strain energy can properly be calculated only for mixtures of monoatomic substances, its contribution to the enthalpy of segregation is always negative [26], indicating that the segregation of the excess Zn atoms towards the surface or the grain boundaries of the ZnO films might be spontaneous.

In the famous model of segregation proposed by Wynblatt and Ku [46], one of the contributions to the enthalpy of segregation—besides the elastic strain energy–is also the difference between the surface energies of the minority substance (in our case Zn) and the majority substance (in our case ZnO), multiplied by the surface area per atom. As the surface energy of Zn $\gamma_{Zn}$ is below 0.573 J/m${}^{2}$ [47,48,49] and the surface energy of ZnO $\gamma_{ZnO}$ is within 0.94 and 4.05 J/m${}^{2}$ [50,51,52], then the value of $\gamma_{Zn}-\gamma_{ZnO}$ will always be negative. Thus we might assume that segregation of Zn atoms towards the surface or the grain boundaries of the ZnO films is occurring spontaneously.

In the case of samples deposited at room temperature, this process would result in a non-uniform distribution of Zn atoms throughout a sample, with the possibility of Zn precipitations at the ZnO grain boundaries and at the film’s surface. The presence of diffraction peaks at 39${}^{\circ }$ and 43${}^{\circ }$ corresponding to 100 and 101 surfaces of Zn crystallites (see Figure 4), confirms the presence of such precipitates. Moreover, the Zn-to-O atomic ratio estimated from the XPS spectra recorded from the surface is 1.89 (see Table 6) which indicates a high excess of Zn atoms with respect to normal ZnO stoichiometry.

The situation is, however, drastically different in the case of samples deposited on substrates at elevated temperatures. The higher the substrate temperature, the higher the probability that the deposited material will grow in an island (Volmer–Weber) mode. A metric that helps determine this is the homologous temperature $T_{h}$ defined as [53,54]:

(7) $$T_{h}=T_{s}/T_{m}$$

where $T_{s}$ is the substrate temperature and $T_{m}$ is the bulk melting temperature of the deposited material. As a threshold, 0.3 is generally accepted as a value above which an island mode is observed [53]—and the higher the value, the longer the island mode persists during the deposition process. As in the case of e-beam evaporated ZnO there is a large excess of Zn atoms due to the decomposition of the zinc oxide, the $T_{m}$ is probably closer to the bulk melting temperature of Zn (693 K) than the bulk melting temperature of ZnO (2247 K). For pure Zn, even the case of the substrate at room temperature, the value of homologous temperature is already as high as 0.425 and for higher temperatures, it will be even greater. Thus, we can assume, that the ZnO films during our deposition process grow in an island mode.

If that is the case, then the surface-to-volume ratio at the initial stages of the growth is very large, and for elevated substrate temperatures it may remain so even in the late stages of the growth. Thus, the excess of Zn atoms might quickly segregate towards the surface of these ZnO islands and form Zn surface precipitations even during deposition. This should progress at a faster rate for elevated substrate temperatures—for instance, it has been demonstrated that elevating the substrate temperature from room temperature to 100 ${}^{\circ }$C results in faster segregation of Ge atoms within Ag films [55]. The Zn surface precipitations are expected to be very thin, not exceeding the thickness of several nm. It has been shown previously that for such thin metal layers, the melting temperature (and thus also the boiling temperature) is much lower than in the case of bulk materials [56]. This suggests that in the case of elevated substrate temperatures, the excess Zn precipitations boil or sublimate from the surface and leave the system thus forming a much thinner, though a more stoichiometric ZnO film. The Zn-to-O atomic ratio, in this case, is closer to the stoichiometric value of 1 (see Table 6) than in the case of samples deposited at room temperature, but still an excess of Zn atoms in XPS measurements is observed. This is probably due to the fact, that the XPS spectra (see Figure 5) are collected from the very surface, to which Zn atoms segregate. The phenomenon described above certainly contributes to the fact, that the thickness of the samples deposited at elevated temperatures are lower than in the case of room temperature (see Table 2), but is probably not the only cause. We believe that, at elevated substrate temperatures, the adsorption energy of Zn and O adatoms might be smaller than their thermal energy, which may result in whole ZnO clusters desorbing from the surface of the substrate, which reduced the overall film thickness. Since the deposition rate of the fabricated samples was very low (0.3 Å/s), the movement of adatoms is not restricted by the stream of subsequent adatoms, which, in a typical case, would prevent such desorption.

The shape of the optical constants of the deposited films (see Figure 2) can be explained taking into account the above-mentioned reason. The metallic-like layers (S_ RT and S_ RT_ 300) consist of both ZnO and Zn crystallites (see Figure 4a). Their optical constants exhibit metallic-like behaviour, however without strong absorption caused by the interaction of electromagnetic radiation with free-carriers. This is due to negligible electrical continuity between zinc crystallites since they are separated by ZnO. The layers deposited at RT and annealed at 500 and 800 ${}^{\circ }$C contain only ZnO crystallites. General shapes of the refractive index and the extinction coefficient curves (see Figure 2b) are close to those determined for ZnO [38], however, the values are significantly lower. In the IR spectral range, *n* equals only 1.5–1.6. Although the band-gap energy was established to be 3.24 eV (value reported for stoichiometric ZnO [4,13]), the value of *k* in the absorbing spectral range (wavelengths below ∼380 nm) is about 0.2 or 0.3 for the S_ RT_ 500C and S_ RT_ 800C specimens, respectively. These discrepancies most probably result from the fact that the ZnO crystallites in these samples grow in different orientations, which also influences the nanoporosity of the fabricated material. The films deposited at 100 ${}^{\circ }$C and 200 ${}^{\circ }$C contain the ZnO crystallites with almost exclusively the 002 direction (see Figure 5b,c) and their optical constants, as well as band-gap energy (3.19 eV), are close to the values reported for the stoichiometric zinc oxide [4,13]. The Zn-rich extremely thin surface film does not affect the optical constants of the deposited ZnO layers. It should be noted that annealing of ZnO films deposited at 100 and 200 ${}^{\circ }$C leads to the increase in the mean crystallite size (see Table 4 and Figure 4d) for the 002 direction from 18–19 nm to 30–31 nm. This effect causes the slight blue-shift of band-gap energy from 3.19 to 3.21–3.22 eV (see Table 3).

## 5. Conclusions

Zinc oxide films were produced by means of the electron beam evaporation method and investigated using atomic force microscopy, scanning electron microscopy, powder X-ray diffraction, X-ray photoelectron spectroscopy and spectroscopic ellipsometry techniques. Influence of substrate and annealing temperatures on the film composition as well as microstructure and optical properties was examined. We have shown that decomposition of ZnO during evaporation and segregation of Zn atoms during evaporation and post-deposition annealing significantly affect the real part of the complex refractive index and the extinction coefficient. We have shown that zinc oxide film produced at room temperature consists of ZnO and Zn crystallites and its optical constants exhibit a metallic-like behaviour. Annealing at 500 and 800 ${}^{\circ }$C leads to desorption of Zn atoms form the surface during deposition. As a result, we have obtained a layer with only ZnO crystallites which is characterised by a complex refractive index typical for semiconducting materials. However, because of a variety of orientations in which the crystallites have grown there is an increased volume of grain boundary voids. Thus, the values of the refractive index are significantly lower (1.5–1.7 in the visible spectral range) than those reported for high-quality zinc oxide films. In the case of the ZnO layers deposited at 100 and 200 ${}^{\circ }$C are characterised by an order of magnitude smaller thickness than that obtained for films evaporated at RT. The real part of the complex refractive index exhibits high value (1.9) in the non-absorbing spectral range and annealing does not lead to significant changes in the optical constants of the fabricated zinc oxide layers. The value of the band-gap energy for all the films exhibiting semiconducting behaviour was established to be ∼3.2 eV.

## Figures and Tables

**Table 1:** Temperatures of deposition ($T_{d}$) and annealing ($T_{a}$) for the investigated samples.

Sample id.	$T_{d}$ (${}^{\circ }$C)	$T_{a}$ (${}^{\circ }$C)
S_RT	RT	–
S_RT_300C	RT	300
S_RT_500C	RT	500
S_RT_800C	RT	800
S_100C	100	–
S_100C_300C	100	300
S_100C_500C	100	500
S_100C_800C	100	800
S_200C	200	–
S_200C_300C	200	300
S_200C_500C	200	500
S_200C_800C	200	800

**Table 2:** The form of the complex refractive index used to parametrise optical constants of the produced films, thicknesses of ZnO ($d_{ZnO}$) and rough ($d_{r}$) layers as well as roughness parameters ($R_{a}$, $R_{q}$ and $R_{max}$) determined for the investigated samples.

Sample id.	Form of $\tilde{n}$	$d_{\mathrm{ZnO}}$ (nm)	$d_{r}$ (nm)	$R_{a}$ (nm)	$R_{q}$ (nm)	$R_{\max}$ (nm)
S_RT	Equation (3)	325 ± 10	37.2 ± 1.3	20.7	16.6	150
S_RT_300C	Equation (3)	299 ± 20	41.3 ± 1.5	19.0	14.9	151
S_RT_500C	Equation (4)	266.0 ± 0.3	40.6 ± 0.2	19.4	15.4	147
S_RT_800C	Equation (4)	199.5 ± 0.5	28.3 ± 0.4	19.5	15.2	161
S_100C	Equation (4)	37.6 ± 0.1	7.5 ± 0.2	1.5	1.2	11.5
S_100C_300C	Equation (4)	38.5 ± 0.2	6.9 ± 0.3	2.3	1.7	18.7
S_100C_500C	Equation (4)	38.3 ± 0.2	7.5 ± 0.3	1.7	1.2	26.9
S_100C_800C	Equation (4)	38.2 ± 0.2	5.4 ± 0.3	2.6	1.9	32.4
S_200C	Equation (4)	36.5 ± 0.2	6.7 ± 0.2	1.5	1.2	11.3
S_200C_300C	Equation (4)	36.7 ± 0.3	5.9 ± 0.3	1.9	1.5	16.0
S_200C_500C	Equation (4)	37.1 ± 0.3	6.5 ± 0.4	2.1	1.7	15.6
S_200C_800C	Equation (4)	36.6 ± 0.2	5.8 ± 0.3	2.8	2.2	21.9

**Table 3:** The band-gap energy ($E_{g}$) estimated for the deposited films.

Sample id.	$E_{g}$ (eV)	$E_{g}$ (nm)
S_RT	- ${}^{a}$	- ${}^{a}$
S_RT_300C	- ${}^{a}$	- ${}^{a}$
S_RT_500C	3.239 ± 0.002	382.8 ± 0.3
S_RT_800C	3.238 ± 0.003	382.9 ± 0.4
S_100C	3.191 ± 0.001	388.5 ± 0.2
S_100C_300C	3.194 ± 0.001	388.2 ± 0.2
S_100C_500C	3.206 ± 0.001	386.7 ± 0.2
S_100C_800C	3.219 ± 0.001	385.2 ± 0.2
S_200C	3.191 ± 0.002	388.5 ± 0.3
S_200C_300C	3.191 ± 0.002	388.5 ± 0.3
S_200C_500C	3.201 ± 0.002	387.2 ± 0.3
S_200C_800C	3.214 ± 0.002	385.8 ± 0.3
${}^{a}$ the band-gap energy has not been determined.		

**Table 4:** The average Zn and ZnO crystallite size (<D>) estimated based on the Scherrer formula.

	<D> (nm)						
Sample id.	Zn (100)	Zn (101)	Zn (102)	ZnO (100)	ZnO (002)	ZnO (101)	ZnO (110)
S_RT	13 ± 2	13 ± 2	9 ± 3	6 ± 4	9 ± 5	9 ± 5	9 ± 3
S_RT_300C	13 ± 2	10 ± 3	9 ± 3	9 ± 5	11 ± 3	9 ± 5	9 ± 4
S_RT_500C	- ${}^{a}$	- ${}^{a}$	- ${}^{a}$	15 ± 3	13 ± 2	11 ± 2	12 ± 4
S_RT_800C	- ${}^{a}$	- ${}^{a}$	- ${}^{a}$	25 ± 3	22 ± 2	21 ± 2	12 ± 4
S_100C	- ${}^{a}$	- ${}^{a}$	- ${}^{b}$	- ${}^{a}$	19 ± 2	- ${}^{b}$	- ${}^{b}$
S_100C_300C	- ${}^{a}$	- ${}^{a}$	- ${}^{b}$	- ${}^{a}$	19 ± 2	- ${}^{b}$	- ${}^{b}$
S_100C_500C	- ${}^{a}$	- ${}^{a}$	- ${}^{b}$	- ${}^{a}$	26 ± 2	- ${}^{b}$	- ${}^{b}$
S_100C_800C	- ${}^{a}$	- ${}^{a}$	- ${}^{b}$	- ${}^{a}$	31 ± 2	- ${}^{b}$	- ${}^{b}$
S_200C	- ${}^{a}$	- ${}^{a}$	- ${}^{b}$	- ${}^{a}$	19 ± 3	- ${}^{b}$	- ${}^{b}$
S_200C_300C	- ${}^{a}$	- ${}^{a}$	- ${}^{b}$	- ${}^{a}$	18 ± 2	- ${}^{b}$	- ${}^{b}$
S_200C_500C	- ${}^{a}$	- ${}^{a}$	- ${}^{b}$	- ${}^{a}$	25 ± 2	- ${}^{b}$	- ${}^{b}$
S_200C_800C	- ${}^{a}$	- ${}^{a}$	- ${}^{b}$	- ${}^{a}$	30 ± 2	- ${}^{b}$	- ${}^{b}$
${}^{a}$ not detected; ${}^{b}$ negligible intensity.							

**Table 5:** Concentration of detected elements (Zn, O and C) at the surface of the produced layers as well as the ratio of Zn to O atoms.

Sample id.	C${}_{\mathrm{Zn}}$ (%)	C${}_{O(\mathrm{Zn})}$ (%)	C${}_{O(\mathrm{vac}.)}$ (%)	C${}_{O(\mathrm{ads}.)}$ (%)	C${}_{O(\mathrm{tot}.)}$ (%)	C${}_{C(\mathrm{tot}.)}$ (%)
S_RT	44.23	25.28	16.16	1.84	43.28	12.49
S_RT_300C	48.19	29.15	9.67	2.65	41.47	10.34
S_RT_500C	51.78	33.89	4.35	0.70	38.94	9.28
S_RT_800C	59.43	37.64	1.72	0.79	40.15	0.42
S_100C	43.23	25.99	12.00	3.95	41.94	14.83
S_100C_300C	43.82	29.46	5.80	6.34	41.60	14.58
S_100C_500C	55.60	35.99	1.64	1.25	38.88	5.52
S_100C_800C	58.50	36.19	2.90	1.28	40.37	1.13
S_200C	46.98	28.09	9.93	3.16	41.18	11.84
S_200C_300C	48.08	30.30	8.26	1.70	40.26	11.66
S_200C_500C	56.41	34.67	2.04	0.24	36.95	6.64
S_200C_800C	60.45	33.64	4.82	0.48	38.94	0.61

**Table 6:** The estimated ratio of Zn to O atoms.

Sample id.	Zn:O (tot.)	Zn:O (Zn)
S_RT	1.07	1.75
S_RT_300C	1.24	1.65
S_RT_500C	1.35	1.53
S_RT_800C	1.51	1.58
S_100C	1.14	1.66
S_100C_300C	1.24	1.49
S_100C_500C	1.48	1.54
S_100C_800C	1.50	1.62
S_200C	1.24	1.67
S_200C_300C	1.25	1.59
S_200C_500C	1.54	1.63
S_200C_800C	1.57	1.80
